# Optimization of Transcription Factor-Driven Neuronal Differentiation from Human Induced Pluripotent Stem Cells for Disease Modelling and Drug Screening

**DOI:** 10.1007/s12015-025-10845-4

**Published:** 2025-01-31

**Authors:** Martina Servetti, Martino Caramia, Giulia Parodi, Fabrizio Loiacono, Ennio Nano, Giorgia Biddau, Lorenzo Ferrando, Lisastella Morinelli, Pierluigi Valente, Sergio Martinoia, Andrea Escelsior, Gianluca Serafini, Serena Tamburro, Simona Baldassari, Anna Fassio, Fabio Benfenati, Anna Corradi, Bruno Sterlini

**Affiliations:** 1 Center for Synaptic Neuroscience and Technology, Istituto Italiano di Tecnologia, Largo Rosanna Benzi 10, 16132 Genova, Italy; 2 Dipartimento di Medicina Sperimentale, Università di Genova, Viale Benedetto XV, 3, Genova, 16132 Italy; 3 IRCCS Ospedale Policlinico San Martino, Largo Rosanna Benzi 10, Genova, 16132 Italy; 4 Department of Informatics, Bioengineering, Robotics, and Systems Engineering (DIBRIS), University of Genova, Genoa, Italy; 5 Dipartimento di Matematica, Università di Genova, Genova, Italy; 6 Unit of Medical Genetics, IRCCS Istituto Giannina Gaslini, Genoa, Italy; 7 Department of Neurosciences, Rehabilitation, Ophthalmology, Genetics, Maternal and Child Health (DiNOGMI), University of Genoa, Genoa, Italy

**Keywords:** Transcription factor–driven differentiation, iGluNeurons, iPSCs, NGN2-mediated neuronal differentiation, Electrophysiological recordings, FACS sorting

## Abstract

Progress of human brain in vitro models stands as a keystone in neurological and psychiatric research, addressing the limitations posed by species-specific differences in animal models. The generation of human neurons from induced pluripotent stem cells (iPSCs) using transcription factor reprogramming protocols has been shown to reduce heterogeneity and improve consistency across different stem cell lines. Despite notable advancements, the current protocols still exhibit several shortcomings. This study focuses on standardizing and optimizing the procedure for iPSC-derived glutamatergic neurons generation through the inducible overexpression of Neurogenin-2. Noteworthy refinements include stringent scrutiny of genomic rearrangements post-fibroblast reprogramming, selection of a homogeneously integrated NGN2-cassettes population, and the incorporation of an intermediate step during neuronal differentiation to store neuronal progenitors. The neural culture showed a high degree of neuronal maturation and consistency, as shown by single-cell and network electrophysiological recordings. These advancements aim to provide more reliable tools for disease modelling and drug screening in neurological disorders.

**Graphical Abstract:**

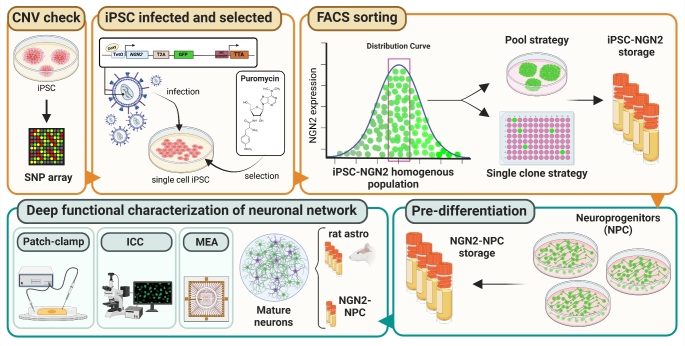

**Supplementary Information:**

The online version contains supplementary material available at 10.1007/s12015-025-10845-4.

## Introduction

The human brain develops as an organ specialized for intricate, higher-order computational processes, encompassing cognition, memory, emotion, language, and behaviour. These functions are made possible by a series of coordinated, sequential cellular and molecular processes that are governed by genetic blueprints that have been acquired during evolution. Mammals share many developmental processes in the brain, and functional research employing traditional animal models has made enormous strides toward understanding their shared genetic mechanism. Indeed, for many years, primary rodent neurons have been the go-to model for studying mammalian neurobiology. However, evolution has also generated a multitude of species-specific traits that may underpin human-specific higher-order brain functions or dysfunctions [1]. Because of these species-related variations, the use of animal models to explain molecular, cellular, or circuit complexity and forecast human treatment responses is severely hampered; especially for these reasons, human cell-based strategies that may capture unique, human-specific characteristics are needed [2].

Recent advances in induced pluripotent stem cell (iPSC) technologies have overcome some of these difficulties by enabling the differentiation of human iPSCs into populations of human neurons, generating unprecedented access to human neuronal cells in vitro and providing new avenues to study human brain function and disease [3]. iPSC generation has become standardized and efficient, largely due to commercially available kits that have streamlined the process. Two main strategies are used to generate neurons from iPSCs: (i) extrinsic factors to mimic the stepwise processes of neurogenesis, directing cells through different progenitor states and toward distinct cell lineages (such as Dual smad inhibition eventually followed by different morphogens [4, 5]), or (ii) the induced expression of lineage-specifying transcription factors to directly differentiate cells to a specific fate, often bypassing the progenitor stage [6].

Contrasting with extrinsic factors differentiation approaches, transcription factor programming-based protocols significantly expedite neuronal production, demonstrating reduced heterogeneity and enhanced consistency across various stem cell lines [7]. Among these, the most used is Neurogenin 2 (NGN2), a bHLH transcription factor that has received particular attention for its application for the in vitro differentiation of fibroblasts and iPSCs into glutamatergic neurons and, less frequently, in a range of other neuronal subtypes such as dopaminergic, serotonergic, motor, sensory neurons and astrocytes [8]. Thoma's seminal papers in 2012 laid the foundation for this method [9] followed by Sudhof's lab demonstrating the generation of glutamatergic neurons through ectopic NGN2 expression in 2013 [10]. Since then, numerous publications have contributed several improvements to the protocol [6].

However, based on current literature, the standardization of post-reprogramming processes, especially in neuronal differentiation, remains deficient. Indeed, there are numerous shortcomings within the protocols developed to date. One of the main concerns of the current protocols is the extreme variability of the number of copies of NGN2 expressed by the starting iPSC, due to the random infection procedure and leading to differentiated neurons at different levels of maturation [11].

This study aims to standardize and optimize the entire process of generating iPSC-derived glutamatergic neurons (iGluNeurons) through the inducible overexpression of NGN2. Key improvements include: (i) stringent screening for genomic rearrangements from fibroblast reprogramming to iPSC, to avoid selecting iPSCs with undetectable genomic rearrangements by current evaluation methods such as karyotype (ii) selecting a population with homogeneous integration of NGN2 cassettes to reduce heterogeneity caused by variable NGN2 expression levels, and (iii) incorporating an intermediate step during neuronal differentiation to store neuronal progenitors, thereby facilitating their use across multiple experiments and reducing variability. These improvements achieved a highly robust and reproducible method for generating iGluNeurons.

## Results

### iPSC Generation: from Fibroblasts to Post-reprogramming Quality Controls

In recent years, several biobanks have emerged to respond to the demand of human iPSCs for research. This network of biobanks has led to the formulation of guidelines, both in legal and laboratory aspects with particular emphasis on post-reprogramming quality control [12, 13]. Indeed, the use of low-quality iPSCs not only squanders research resources, but also jeopardizes publications, renders patient’s samples useless and hampers the potential clinical and therapeutic advancements stemming from iPSC research.

**Fig. 1:**
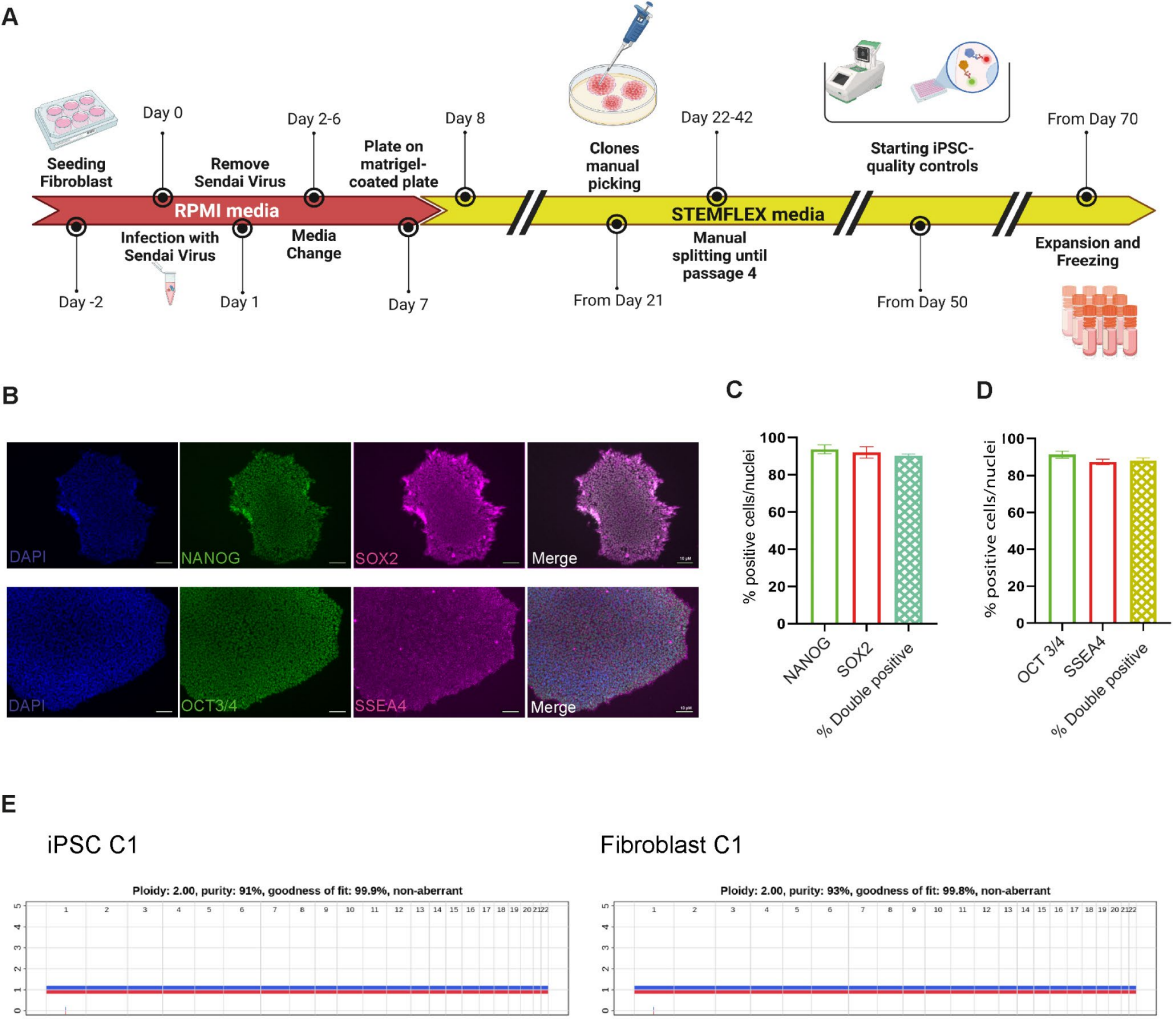
Generation and characterization of iPSCs from fibroblasts. (**A**) Timeline of the generation of iPSCs, starting from the manual picking of emerging colonies after virus infection, further expansion, and full characterization. (**B**) Representative immunostaining of the undifferentiated state markers NANOG, SOX2, OCT3/4, and SSEA4 (scale bar 50 µM). (**C**, **D**) Images from 4 cell lines were quantified using ImageJ for colocalization of nuclear and surface markers (expressed as percentages of DAPI-positive nuclei; means ± SEM). (**E**) Comparison of SNP array results between fibroblasts and iPSCs reprogrammed from the same cell line

The generation of iPSCs from fibroblasts that will be used along the study, and the quality controls conducted in line with the recently formulated guidelines [12, 13] are depicted in Fig. 1A. We generated iPSC cell lines (C4, P1, P2, P3) using Sendai kit 2.0: fibroblasts were infected with Sendai Viruses. After 3 weeks, when colonies start to appear, we manually picked between 10 to 15 clones per each cell line. Single clones were split until passage 5 and then quality controls were performed, including RT-PCR to check the Sendai virus vector loss and immunostaining for the standard undifferentiated state markers NANOG, SOX2, OCT3/4, SSEA4 (Fig. 1B-D and Table 1) [14]. Additionally, our focus was directed towards evaluating genomic rearrangements. As previously pointed out, the limited resolution of conventional methods, such as array-CGH and karyotyping, fails to detect many genomic rearrangements [15] and this limitation introduces potential confounding factors. To address the need for higher resolution in verifying genomic rearrangements, we employed the Single Nucleotide Polymorphism (SNP) Infinium array that have 560,000 probes all over the genome for comparing the iPSC clones to the respective fibroblasts. The Copy number variation (CNV) screening analysis did not reveal newly acquired genomic aberrations in any of the iPSC lines compared to fibroblast cell lines of origin, as shown in Fig. 1E for an individual cell line. The ASCAT R package was run with different penalty parameters (from 70 to 150 by 10) and the results were coherent. Thus, our reprogramming protocol generated pluripotent and genomically stable iPSCs, making them eligible for further manipulations.

**Table 1:** iPSC information and quality control

ID	Subjects Information			Quality control			Ref.
	Age	Gender	Source	Sendai virus	Undifferentiated state markers	CNV Check	
C1	young	F	Fibroblast	✓	✓	SNP array	**/**
C2	young	F	Fibroblast	✓	✓	SNP array	[16]
C3	adult	M	Fibroblast	✓	✓	Array-CGH	[17]
C4	young	M	Fibroblast	✓	✓	SNP array	[18]
P1	young	M	Fibroblast	✓	✓	SNP array	[18]
P2	young	M	Fibroblast	✓	✓	SNP array	[18]
P3	young	M	Fibroblast	✓	✓	SNP array	[18]

*Left:* IPSC cell line information about age, gender and source of cells. *Right:* Quality controls performed on iPSC clones (from left: RT-PCR for Sendai virus, undifferentiated state markers (NANOG, SOX2, OCT3/4, SSEA4), copy number variations assay)

### Generation of iPSC Population with Homogenous Expression of NGN2

The generation of glutamatergic neurons through the enforced expression of NGN2 has become widely employed, primarily due to the limitations associated with extrinsic factor-driven neuronal differentiation methods. The latter approach is hindered by time-consuming procedures, involving complex multi-step processes, and often yields functionally variable neurons [19].

Despite the widespread adoption of NGN2 in the last few years, a recent study has highlighted the presence of significant heterogeneity within the resulting neuron population across diverse cell lines. The variable expression levels of the reprogramming factor NGN2 within the population, as highlighted by Treutelein’s group, may contribute to the observed heterogeneity [11]. Despite precautions ensuring uniform lentivirus transgene delivery among distinct iPSC lines are taken, e.g., by using identical virus titers and maintaining a similar post-infection passage number at day 0 of differentiation, challenges persist.

**Fig. 2:**
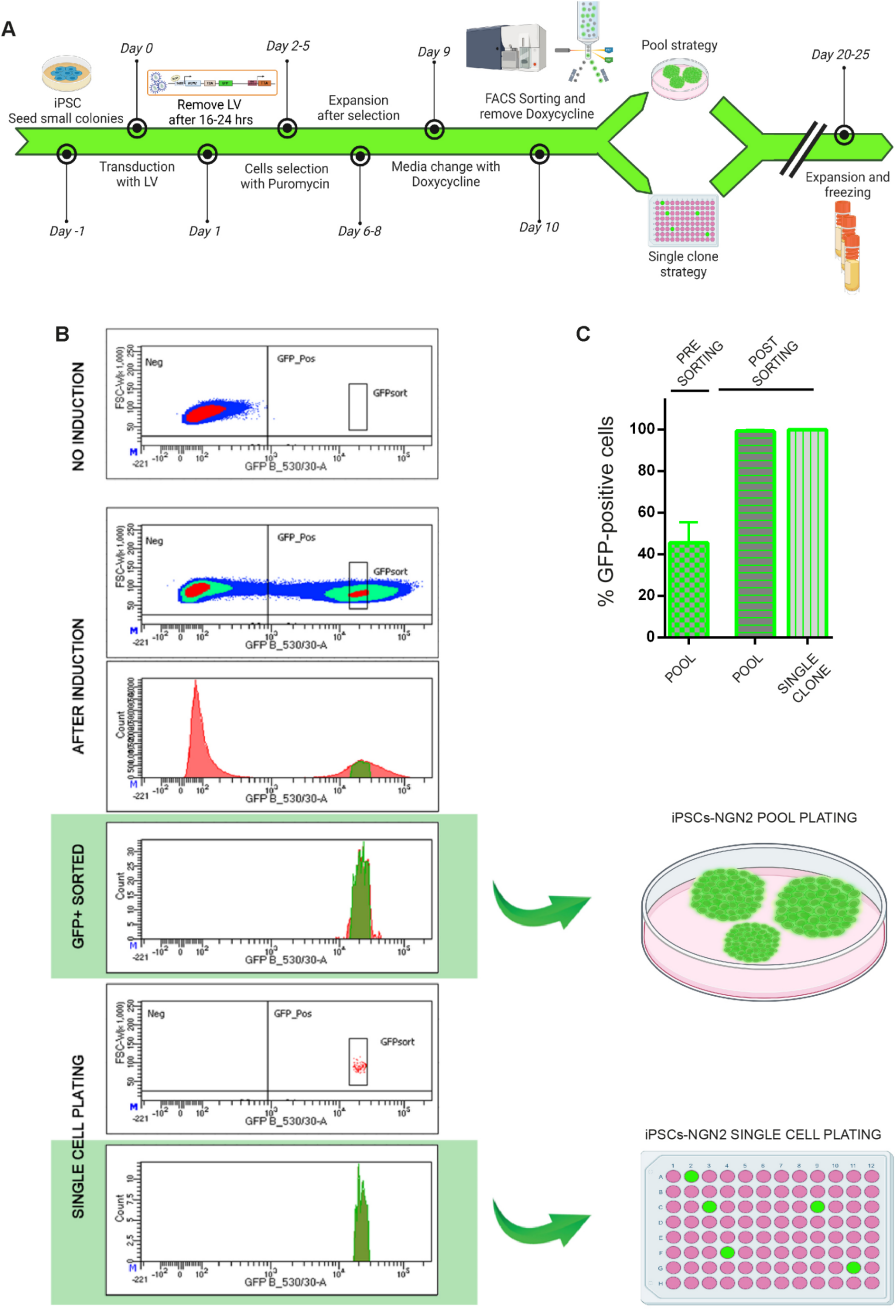
Lentiviral infection with “*all-in-one Tet-on”-*vector NGN2 and FACS sorting of iPSC-NGN2 positive cells. (**A**) Timeline of the lentiviral infection with "*all-in-one Tet-on”* vector NGN2 and subsequent FACS sorting of cells. Briefly, after infection and selection, NGN2 and GFP expression were transiently induced and, the next day, GFP-positive cells were sorted and plated either as a pool or as single clones. (**B**) Representative dot plots of a single cell line showing the various steps of FACS sorting, starting from non-induced cells to single-cell seeding. These plots highlight how the selected cell region corresponds to the largest cell population exhibiting the same fluorescence intensity (middle panels). (**C**) Histograms of the percentage of GFP-positive cells before and after sorting, comparing pool and single clone strategies for all 7 IPSC lines (4 iPSC lines from healthy controls and 3 iPSC from patients carrying a mutation in the *PRRT2* gene) tested along the study. Data are represented as means ± SEM

To address this issue, we used a vector in which NGN2 is linked to GFP through a T2A sequence and chose to isolate a subpopulation of iPSCs exhibiting both median and homogeneous expression of GFP (and thereby of NGN2) by Flow-activated cell sorting (FACS) sorting (Fig. 2A).

Our methodology involved lentiviral transduction of iPSCs using the “*all-in-one Tet-on”* vector generated by the Gage’s group [20] to express rtTA and the NGN2/GFP transcript under the control of the Tetracycline response element promoter (TREtight promoter). Following infection, iPSCs underwent puromycin selection for 4 days, allowing subsequent expansion to perform FACS sorting. As can be observed in the top panel of Fig. 2B, the transduced and resistance-selected iPSCs do not exhibit any fluorescence, demonstrating well-controlled expression by TREtight promoter without any leakage. Upon induction with tetracycline, a significant proportion of cells ranging from 30 to 80% appear negative for GFP, while the positive ones exhibit a strong heterogeneity in GFP expression (Fig. 2C). As most differentiation protocols described in literature employ these cells as a starting point for differentiation, a significant heterogeneity in neuronal populations can be expected [11, 21].

In our experimental protocol (Fig. 2A), after a 12-h induction with doxycycline, we isolated the subpopulation exhibiting a consistent median level of GFP-NGN2 expression by FACS sorting (“GFPsort” gate as shown in the density plot of Fig. 2B; see Supplementary Fig. 1A, B for gating strategy).

These cells were then plated in Stemflex media, without doxycycline, on Petri dishes to expand iPSC pools. A portion of the pooled samples were subjected to a second round of sorting, wherein individual cells were plated into 96-well plates to generate distinct single clones (Fig. 2B). This approach allows to differentiate neurons either starting from iPSC pools or single clones. Moreover, this versatile step can be extended to other transcription factor-based protocols or to the two-plasmid strategy (Tta and TREpromoter/transcription factor localized in two distinct vectors) [22].

The sorted iPSCs that have integrated the *'all-in-one Tet-on'* cassettes into their genome (iPSC-NGN2) took approximately 7 to 10 days to form colonies of medium size and then were subsequently split using Versene. The maintenance of pluripotency in these IPSC-NGN2, despite the brief NGN2 expression, was confirmed through undifferentiated state markers staining (Supplementary Fig. 1C, D). After five passages of iPSC-NGN2, we induced the cells and verified the absence of any leakage and silencing of the integrated cassettes. Flow cytometry analysis demonstrates that prior induction, all cells were negative; post-induction, almost all cells displayed a positive signal, maintaining consistent intensity levels (Supplementary Fig. 1E, F). This crucial step allowed us to store a substantial number of cells at the same passage with comparable integration of the *'all-in-one Tet-on*' cassette, increasing the intra- and inter-experimental homogeneity.

### iPSCs-NGN2 Differentiation to iGluNeurons

**Fig. 3:**
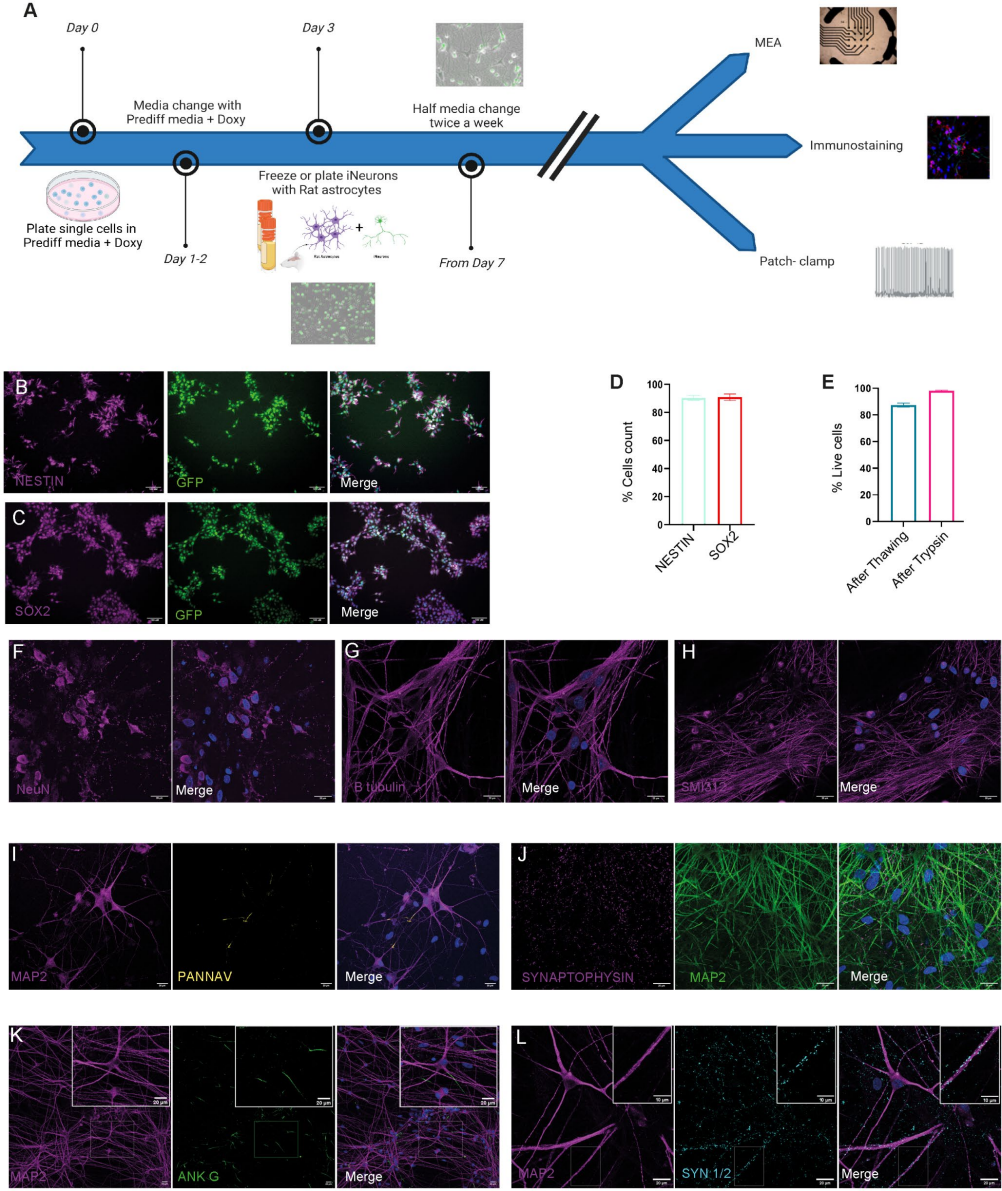
IPSCs NGN2 pre-differentiation and differentiation steps. (**A**) Schematic protocol to differentiate iPSCs into iGluNeurons. Cells were pre-differentiated for 3 days, then cryopreserved. iGluNeurons were generated by plating pre-differentiated cells with rat astrocytes. (**B**, **C**) Representative immunostaining of neuroprogenitor cells using Nestin (**B**) and SOX2 (**C**) markers. (**D**) SOX2- and Nestin-positive cells expressed in percent of DAPI-positive nuclei for all IPSC lines tested. (**E**) Percentage of viability of neuroprogenitors after thawing and trypsinization. (**F-L**) Representative immunostaining of iGluNeurons performed between DIV 35 and DIV 49 for mature neuronal markers: NeuN (**F**), Tubulin beta III (**G**), SMI312 (**H**), MAP2 and NaV channels (**I**), synaptophysin and MAP2 (**J**), AnkyrinG and MAP2 (**K**), Synapsins I/II and MAP2 (**L**) (scale bar 10 µM)

To differentiate iGluNeurons, we employed a two-step protocol based on previously published methods [23, 24] (Fig. 3A). The initial phase, termed the "pre-differentiation step" (pre-diff step), spans 3 days and involves the induction of NGN2 with doxycycline in a medium supplemented with neurotrophic factors (induction media).

As iPSCs grow in colonies, an initial single-cell dissociation step is crucial prior to doxycycline treatment to ensure an optimal environment for subsequent neuronal differentiation. iPSCs were plated in Induction Media, bypassing direct utilization of the Neurobasal medium designed for mature neurons and less suited for the transition from iPSCs to neuroprogenitors (NPC). Instead, DMEM/F12, a nutrient-rich medium widely acknowledged for iPSC cultures, was chosen to provide an ideal milieu for this phase. Over a 3-day in vitro (DIV), a progressive morphological transformation towards the neuroprogenitor fate (characterized by neurites elongation and arborization) was observed (Fig. 3A). By DIV 3, cells uniformly exhibited the typical neuron-like morphology and stained positive for neuroectodermal stem cell markers including NESTIN and SOX2 (Fig. 3B, C) [25, 26]. We found that most of the treated cells were positive for these markers, indicating that almost all treated iPSCs differentiated into NPC (Fig. 3D). At this stage, NPC cells could be cryopreserved as large batches. Overall, this scalable protocol for the generation of cryopreserved batches of NPCs provides a high level of standardization for subsequent analysis.

Subsequently, as described in the timeline of Fig. 3A, NGN2-NPCs were thawed and plated together with rat astrocytes, with an average post-thaw viability of about 90% (Fig. 3E). Without astrocyte support, NPCs can survive only up to DIV12-14, an insufficient time for achieving adequate neuronal maturation (data not shown). Cells were plated at a density of 1,200 cells/mm^2^ [22] comprising 800 neurons and 400 rat astrocytes. The maturation status was evaluated by immunocytochemistry for several neuronal markers. Starting from 4–5 weeks of differentiation, iGluNeurons exhibited markers typical of mature neurons such as (Fig. 3F-L): presynaptic specialization (synaptophysin and synapsins I/II), action initial segment (ANKG and voltage-gated sodium channel, NaV), dendritic processes (MAP2), neuronal post-mitotic nuclear protein (NeuN), vesicular glutamate transporter 1 (vGlut1, supplementary Fig. 2A) and axons (SMI312) [18, 27–29]).

### Functional Characterization iGluNeurons at the Single-Cell Level

**Fig. 4:**
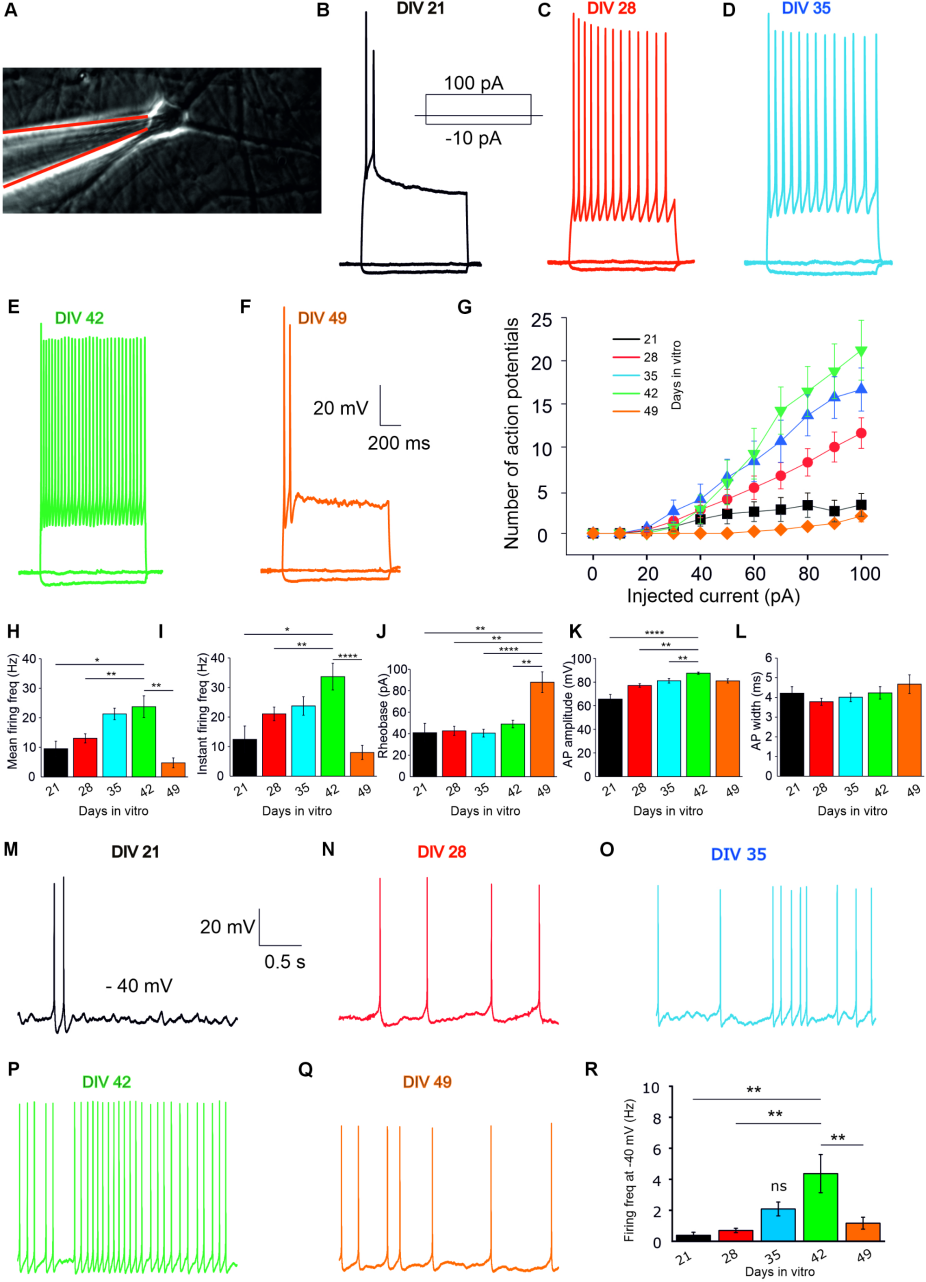
Action potential firing activity of individual iGluNeurons. Action potential firing activity in iGluNeurons. (**A**) iGluNeuron probed by patch pipette. (**B-F**) In vitro whole cell current clamp recording of the action potential firing activity elicited with the injection of depolarizing currents at five timepoints of in vitro differentiation: DIV 21 (**B**), 28 (**C**), 35 (**D**), 42 (**E**) and 49 (**F**). (**G**) Plot of the number of action potentials evoked (y axis) vs the amount of depolarizing current linearly injected (x axis). (**H-L**) Quantification of the main action potential parameters evaluated at each timepoint of differentiation. Mean firing frequency (Hz; **H**), Instantaneous firing frequency (Hz; **I**), rheobase (pA; **J**), action potential amplitude (mV; **K**), action potential width (ms; **L**). (**M-Q**) In vitro whole cell current clamp recording of the action potential spontaneous activity recorded at the subthreshold potential of −40 mV at each timepoint. (**R**) Bar plot showing the mean spontaneous firing frequency of iGluNeurons at each timepoint (Hz). *N* = 3 iPSC cultures from 3 healthy subjects, in 3 independent experiments (an average of *n* = 15 across all time points). Data are expressed as Mean ± SEM. *P < 0.05; **P < 0.01; ***P < 0.001; ****P < 0.0001

To test whether the differentiation of iPSCs in iGluNeurons was effective, we performed whole-cell patch-clamp recordings (Fig. 4A) both in current- and voltage-clamp configurations at various experimental timepoints corresponding to five stages of in vitro differentiation (DIV 21, 28, 35, 42, 49). We first determined whether the injection of a linear gradient of depolarizing current was able to elicit action potential (AP) firing in iGluNeurons in current-clamp recordings. Interestingly, injection of 100 pA depolarizing current was able to evoke AP firing that gradually increased at each timepoint (Fig. 4B-E), although a dramatic reduction in the number of evoked APs was found at DIV 49 and DIV 56 (Fig. 4F, supplementary Fig. 2F). In iGluNeurons, the number of APs (Fig. 4G), as well as the mean and instantaneous firing frequency (Fig. 4H, I) reached the maximum values at DIV 42, followed by a strong decay at DIV 49. The AP amplitude progressively increased from DIV 21 to DIV 42 in the absence of significant changes in the rheobase and AP width (Fig. 4J-L). The spontaneous firing of iGluNeurons was evaluated at each timepoint by bringing neurons at the AP threshold (Vholding −40 mV) by the injection of a constant depolarizing current. Similarly, to what described above, the spontaneous AP frequency was higher at DIV 42 compared to the other timepoints with a strong decrease at DIV 49 and DIV 56 (Fig. 4M-R, supplementary Fig. 2G).

**Fig. 5:**
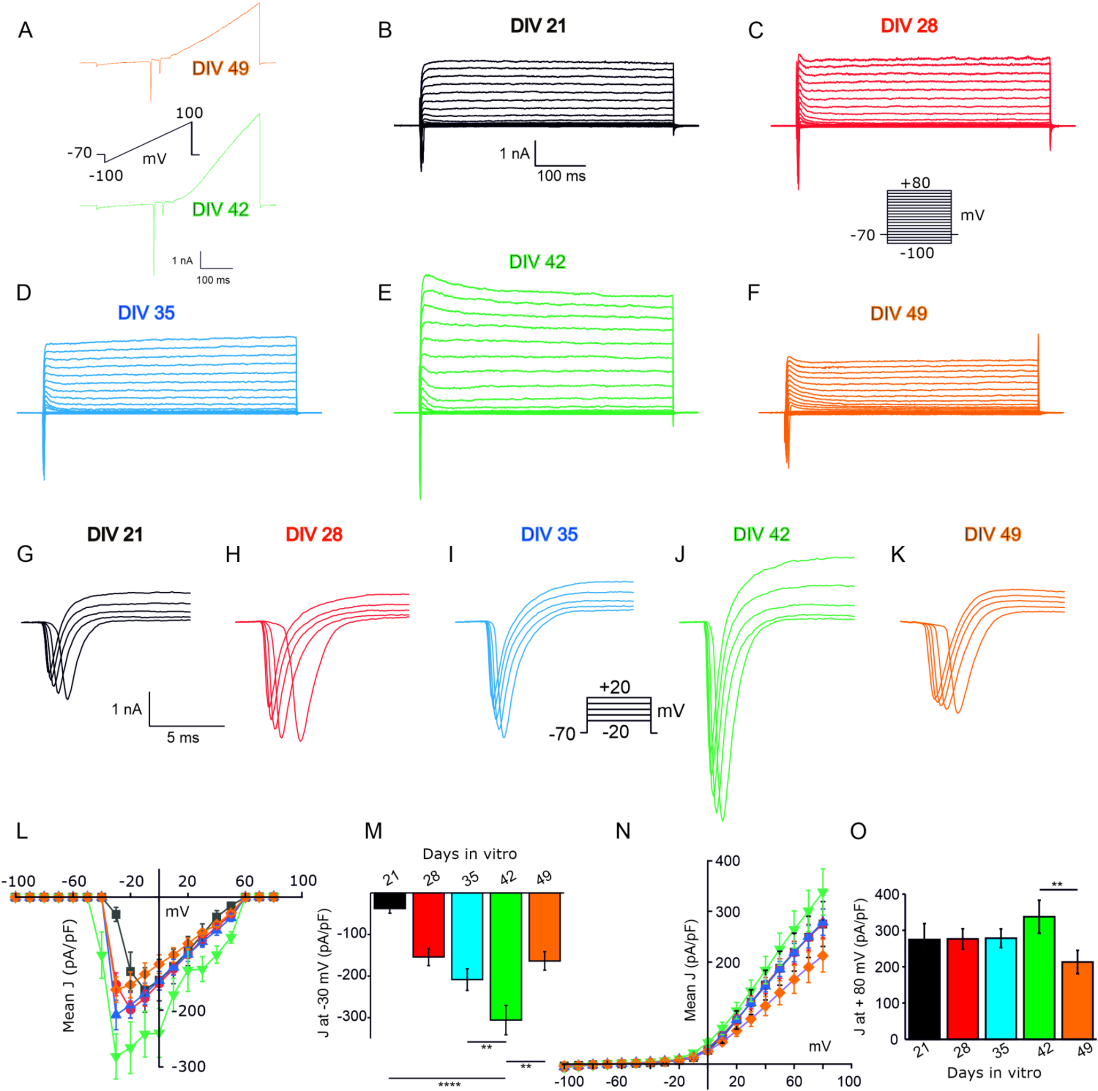
Voltage-gated sodium currents expression in iGluNeurons. (**A**) Exemplificative ramp protocol recorded from −100 mV to + 100 mV, (Vholding = −70 mV, duration of 500 ms) by iGluNeuron at 42 (green) and 49 (orange) DIV (**B-F**) Exemplificative I-V relationships recorded from −100 to + 80 mV (Duration of 500 ms, Vholding = −70 mV, Δ = 10 mV) by iGluNeurons at different timepoints of in vitro differentiation. (**G-K**) Representative whole cell current families recorded from iGluNeurons at each timepoint of in vitro differentiation previously described. As before, cells were clamped at −70 mV. (**L-M**) Mean I-V relationship of the inward (**L**) and outward (**M**) current densities J (pA/pF) at each voltage step obtained in each experimental timepoint. (**N-O**) Bar plot showing the current density measured at −30 (**N**) and + 80 mV (**O**) in each experimental condition. *N* = 3 iPSC cultures from 3 healthy subjects, in 3 independent experiments (an average of *n* = 15 for each time points). Data are expressed as Mean ± SEM. *P < 0.05; **P < 0.01; **P < 0.001; ****P < 0.0001

Single cell excitability was further studied by measuring, in voltage-clamp configuration, inward and outward macroscopic currents elicited by the application of either a depolarizing ramp or depolarizing steps from −100 to + 80 mV (500-ms duration, V holding −70 mV). Both the voltage ramp protocol (Fig. 5A) and the depolarizing step protocol (Fig. 5B-F) performed over the differentiation timepoints, elicited fast-inactivating inward and sustained outward currents that peaked at DIV 42 and decreased in amplitude at DIV 49 and DIV 56. To better assess the inward current, a shorter (50 ms) family of depolarizing voltage steps was used (Fig. 5G-K, Supplementary Fig. 2H).

To build-up I/V curves for inward and outward currents (Fig. 5L, N respectively), current density values (J; pA/pF) were calculated as the ratio between the macroscopic current and the cell capacitance. Inward current densities recorded at −30 mV progressively increased from DIV 21 with a peak at DIV 42 (−305.6 ± 35.32 pA/pF; *n* = 10) that was significantly different from the corresponding values recorded at DIV 35 (−208.32 ± 26.12 pA/pF; *n* = 16) and 21 (37.56 ± 1.11 pA/pF; *n* = 10) (Fig. 5M). On the contrary, no significant differences in the outward current density measured at the current steady state at + 80 mV were found across the timepoints of differentiation up to DIV42 (Fig. 5O). Significant drops in both inward and outward current densities were observed at DIV 49 compared to DIV 42 (Fig. 5M, O). Taken together, these data indicate that iGluNeurons gradually enhance their maturation state across the differentiation timepoints.

### Functional Characterization of iGluNeurons at the Network Level

**Fig. 6:**
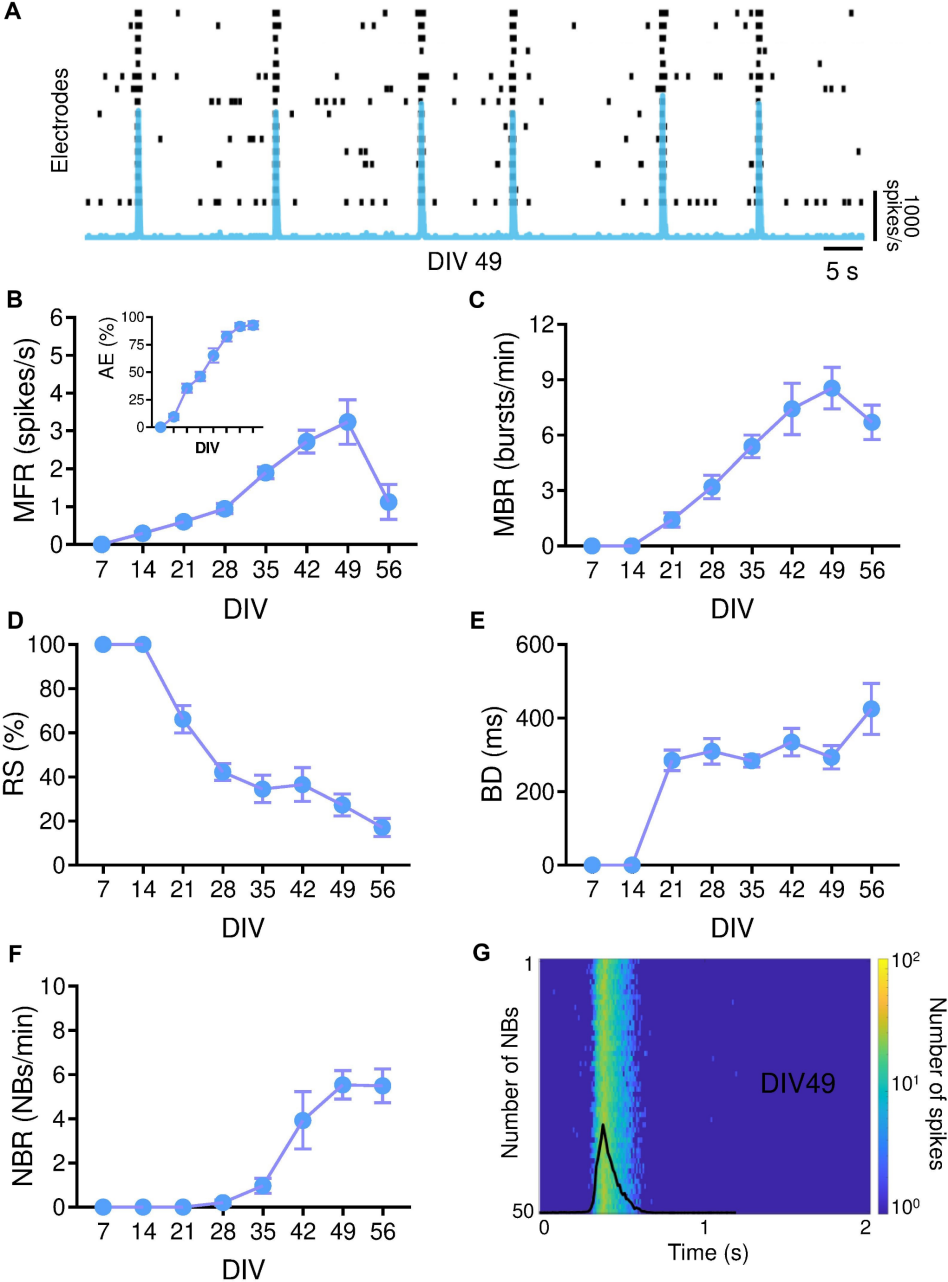
Spontaneous electrophysiological activity of the iGluNeuron network. iGluNeurons were plated on MEA chips. (**A**) Raster plots of 60 s spontaneous activity of a representative neuronal culture at DIV 49 overlapped with the cumulative instantaneous firing rate profile (bin = 10 ms). Spikes are represented by black bars; vertical bands indicate network burst events overlapped with the cumulative instantaneous firing rate profile (bin = 10 ms). (**B-F**) Scatter plots of Mean Firing Rate (MFR; **B**), percentage of Active Electrodes (AE; inset in **B**), Mean Bursting Rate (MBR; **C**), percentage of Random Spikes (RS; **D**), Burst Duration (BD; **E**), and Network Bursting Rate (NBR; **F**). Data are shown as means ± SEM. (**G**) Aligned network bursts with respect to the peak of the longest network burst of a representative network at DIV 49, overlapped with the respective cumulative instantaneous firing rate profile (bin = 10 ms). *N* = 3 iPSC cultures from 3 healthy subjects, in 3 independent experiments (total *n *= 13). The mean values and statistical data are provided in Supplementary Tables 1-3

To study the activity of iGluNeuron networks, we plated iGluNeuron on Micro-electrode array (MEA) devices. Neuronal networks showed sustained firing activity characterized by bursting and network bursting patterns (Fig. 6A). By observing the cultures over development, the number of active electrodes increased during the DIV approaching the 100% of active units at DIV 49 (Fig. 6B, inset). Parallel increases of the firing pattern were observed, reaching maximum values of the mean firing rate (MFR, i.e., the number of spikes in the unit time) at DIV 49 (MFR_DIV49_ = 3.24 ± 1.95 spikes/s, Fig. 6B). A bursting pattern emerged at DIV 21 with the frequency of the bursting events (mean bursting rate—MBR, i.e., the number of bursts per minute) progressively increasing up to DIV 49 (Fig. 6C). The increased bursting was associated with a parallel decrease in random spiking activity (RS, i.e., the number of spikes not belonging to a burst, Fig. 6D), whose values approached 27% at DIV 49. It is worth noting that, at DIV 7 and 14, the neuronal cultures were not characterized by bursting activity (MBR DIV 7,14 = 0 burst/min) and 100% of spikes were classified as random (Fig. 6D). In contrast to their frequency, the duration of the bursts (BD, Fig. 6E) did not show significant changes during the development of the neuronal cultures.

Finally, we evaluated the network bursting activity and, in particular, the frequency of bursting events involving the whole neuronal network. The network bursting rate (NBR, i.e., the number of network bursts per minute, Fig. 6F) showed increasing values as a function of time, reaching a maximum value at DIV 49 (5.55 ± 2.04 NBs/min), in line with the evolution of the firing and bursting patterns. It is interesting to note that despite a detectable bursting pattern occurred at DIV 21, the first network bursting activity only appeared at DIV 28, suggesting that a higher level of network maturation and organization is needed to generate global bursting events.

By considering the electrophysiological activity at the time of the maximum performance of the cultures i.e., at DIV 49, the network burst events, which were aligned with respect to the peak of the longest network burst (Fig. 6G), demonstrated comparable durations and amplitudes, while the average network burst curve (overlapped in black in Fig. 6G) highlighted a canonical shape with a steep rise and a slower decay (see Supplementary Tables 1–3). Finally, we further confirmed the glutamatergic identity of the iGluNeurons through the application of the NMDA receptor antagonist 2-amino-5-phosphonovaleric acid (APV) and the AMPA receptor antagonist 6-cyano-7-nitroquinoxaline-2,3-dione (CNQX). As expected, these treatments significantly reduced the firing activity and altered bursting and network bursting patterns, demonstrating that network activity relies on glutamate-mediated activation of AMPA and NMDA receptors [30]. We also tested the GABA-A receptor antagonist bicuculline (BIC), which had no significant effect on the parameters of the considered neuronal dynamics, further confirming the absence of GABAergic neurons and the exclusively glutamatergic composition of the network (Supplementary Fig. 2B-E). In summary, the network culture reaches the 100% of active electrodes at DIV 49, demonstrating the capability of neurons to successfully adhere to the surface of the MEAs and proving that the neurons were homogeneously functional along the entire surface of the devices. Moreover, the increasing firing pattern observed over time in vitro, which reached its maximum at DIV 49, confirms the progressive maturation of the networks over time. From DIV 21 onwards, neuronal dynamics evolved, self-organizing into more complex and high-frequency events such as bursting and network bursting activities, with network bursts appearing after DIV 28 and RS progressively decreasing. These features, characterized by the progressive development of firing patterns, bursting, and network bursting activity, are characteristic of in vitro cultures of primary neuronal networks.

## Discussion

Our study systematically addressed several shortcomings of current protocols based on the ectopic expression of transcription factors for generating neuronal populations, which are the primary cause of high variability and poor reproducibility in these systems. Specifically, we have focused on reducing variability at multiple levels.

We implemented a much stricter assessment of copy number variations using a 560,000-probe SNP array. This prevented the selection of iPSCs with genetic alterations not detectable by commonly used methods such as karyotyping.

Currently, the protocol described by Frega [22] is the most widely used method for generating glutamatergic iGluNeurons through inducible NGN2 expression [11, 20, 31–34] building upon and improving the original protocol by Zhang [10]. The main advancement introduced by Frega was the creation of iPSC stable integrating Tta and TREpromoter/NGN2 iPSC prior to inducing neuronal differentiation, using the two separate vectors. While this modification improved control over neuronal cell density—critical for ensuring consistency in MEA experiments—it did not fully resolve issues related to population heterogeneity, primarily caused by varying levels of NGN2 expression among cells.

We addressed this limitation by implementing a FACS-based sorting step, which standardizes and homogenizes NGN2 expression within clones of the same pool and across different iPSC-NGN2 lines. This approach effectively eliminated one of the primary sources of variability, reducing heterogeneity in maturation stages and neuronal features. To further minimize inter-experimental variability and enhance control over neuronal density compared to the Frega’s protocol, we introduced a pre-differentiation step that enables cryopreservation of neural progenitors for use in multiple experiments. This approach, as previously demonstrated by several studies [23, 24, 35], facilitates the generation of standardized and reproducible neuronal populations.

Additionally, we incorporated several small refinements to the protocol to further enhance reliability and reproducibility. By employing an “all-in-one Tet-on” vector (containing both tTA and NGN2 under TRE control), we eliminated the need for two antibiotics for selection and infections with two distinct viruses, thereby reducing cellular stress and potential variability. Furthermore, we replaced the murine NGN2 sequence with the human sequence, ensuring greater alignment with the human iPSC model. Finally, to address differentiation variability associated with Fetal bovine serum composition and its batch-to-batch differences, we excluded the 2.5% FBS added after DIV10 in the original protocols [10, 22].

The effectiveness of all these modifications was demonstrated by their consistent application across numerous iPSC lines tested, including those derived from healthy subjects and patients. We observed high levels of maturation and homogeneity in both neuronal marker expression and functional maturation at both single-cell and network levels. Consequently, our system enables the generation of large batches of neuroprogenitors from iPSCs within approximately 20–25 days, suitable for various differentiation experiments with high functional reproducibility. It is of utmost importance that our protocol is adaptable to distinct transcription factors and can be tailored for single-clone approaches, ensuring its versatility and broad applicability across a wide range of experimental contexts and research objectives. The main limitation of this protocol is the random integration of the "all-in-one TetOn" cassette into the genome, compared to other protocols that target safe harbour loci such as adeno-associated virus integration site 1 (AAVS1) [24, 35]. However, generating cell lines with NGN2 with the correct integration into safe harbour loci is a time-consuming step, that greatly reduces the number of lines that can be generated and analysed, whereas our protocol achieves higher integration efficiency, significantly reducing the time required to generate iPSC-NGN2 pools or clones (10–14 days versus 30–42 days) [35].

Finally, thanks to the detailed electrophysiological characterization at both single-cell and network levels, this study can serve as a significant reference point among the numerous existing protocols in the field of iPSC-derived neuron. In this study, single cell patch-clamp recordings demonstrate the successful differentiation of iPSCs to iGluNeurons. iGluNeurons were able to evoke spontaneous firing activity when kept at – 40 mV as subthreshold value of membrane potential. Plus, the injection of a linear gradient of depolarizing current steps successfully elicited AP firing. Importantly, both instantaneous and mean firing frequencies, as well as the AP amplitude were strictly connected to the stage of in vitro differentiation. Accordingly, the fast-inactivating inward currents ascribable to sodium channels responsible for the generation of APs increased throughout in vitro differentiation up to DIV 42, while no significant differences in the sustained outward currents expression were observed. The results of the patch-clamp recordings testify the successful differentiation of iGluNeurons over time. Interestingly, the peak of the firing performance was observed at DIV 42. Longer times in vitro (DIV 49 and DIV 56) brought about a marked drop in the firing activity associated with an increase in the rheobase and a decay in both inward and outward macroscopic currents, indicating that an optimal window for the expression of a full neuronal phenotype exist and that longer times in vitro may be associated with regressive phenomena. Moreover, neuronal networks successfully showed a development characterized by a progressive maturation demonstrated by electrophysiological patterns of activity typical of in vitro cultures of primary neurons, i.e., firing, bursting, network bursting activity, underlining that they can be efficiently used to model both neuronal development and mature functional features in human neurons.

## Conclusion

In conclusion, all the advancements introduced in this protocol compared to previous approaches will enhance standardization and reproducibility, providing a valuable foundation for researchers in the field. Importantly, this methodology can be applied to all neuronal differentiation protocols based on the transcription factor-driven approach. We are confident that the methodology outlined in this study will significantly streamline workflows of studies involving patient-derived neurons, contributing to a deeper understanding of both physiological and pathophysiological mechanisms in the human brain.

## Experimental Procedures

### iPSC Generation from Fibroblast

Healthy iPSC lines (C1, C2, C3) were obtained from the ‘Cell Line and DNA Biobank from Patients affected by Genetic Diseases’ (Istituto G. Gaslini, Genova, Italy), which is a member of the Telethon Network of Genetic Biobanks. iPSCs from affected patients (P1, P2, P3) and one healthy control (C4) were generated, in this study, using the CytoTune™-iPS 2.0 Sendai Reprogramming Kit (Invitrogen, A16518) from dermal fibroblasts. The dermal fibroblasts of the affected patients were derived from three siblings of a consanguineous family segregating the common PRRT2 mutation c.649dupC [18]. The study was approved by the Ethical Committee of regione Liguria. Both patient’s and the controls cells line were generated. The features of iPSC lines are detailed in Table 1.

### iPSC Maintenance

Cells were cultured in Geltrex coated plates (Gibco, A14132-02) and StemFlex medium (Gibco, A3349401). Cells were splitted using Versene Solution (Gibco, 15040066) after 1 × wash of HBSS (Gibco, 14175129) into Stemflex medium with ROCK Inhibitor (RI, Y-27632 dihydrochloride) [1ul/mL]. Freezing solution was prepared using half volume of DMS0 20% (Sigma, D2650) /Fetal Bovine Serum (FBS) (Gibco, 10500064) and half volume of Stemflex medium and ROCK Inhibitor. Briefly, cells were washed once with HBSS, then aspirated HBSS and added Versene solution, kept the cells at 37 °C for 3 min then aspirated the Versene solution and added the Freezing solution. The mycoplasma test is performed routinely.

#### RT-Sendai Virus PCR

Following the CytoTune™-iPS 2.0 Sendai Reprogramming Kit (Invitrogen, A16518) we performed RT PCR on the 4 vectors (See table below for primers). Gene SeV (product size 181 bp). Primers: Forward GGA TCA CTA GGT GAT ATC GAG C*, Reverse: ACC AGA CAA GAG TTT AAG AGA TAT GTA TC*. Gene KOS (product size: 528 bp) Forward: ATG CAC CGC TAC GAC GTG AGC GC Reverse: ACC TTG ACA ATC CTG ATG TGG. Gene Klf4 (410 bp), primers: Forward: TTC CTG CAT GCC AGA GGA GCC C Reverse: AAT GTA TCG AAG GTG CTC AA*. Gene c-Myc (532 bp): Forward: TAA CTG ACT AGC AGG CTT GTC G* Reverse: TCC ACA TAC AGT CCT GGA TGA TGA TG. Housekeeping gene HPRT (5090 bp) Forward: CAA AGA TGG TCA AGG TCG CAA G Reverse: AGT CAA GGG CAT ATC CTA CAA CA. Housekeeping gene: GADPH Forward: TGTGGGCATCAATGGATTTGG Reverse: ACACCATGTATTCCGGGTCAAT. Briefly, cells were washed once with phosphate buffered saline (PBS), then were harvested adding Accutase for 5 min at 37 °C. The cells were collected and centrifuged for 5 min at 500 g. Then the cells pellets were resuspended to eliminate medium residues. After resuspension, the cells were centrifuged again, and the resulting pellets were snap-frozen and stored at at −80 °C. RNA was extracted using the RNeasy Mini kit (QIAGEN; Cat N. 74106). Then we did the retrotranscription using the SuperScript IV (Invitrogen, 18091050). Afterwards we performed a RT-PCR with 2 ul of DNA for 5 reactions per clone (see table in supplementary material). We then ran a 2% Agarose gel at 80 V for 1 h and took gel images.

#### SNP and CNV Analysis

DNA was extracted with the DNeasy Blood & Tissue kit (QIAGEN, 69504) and checked using a spectrophotometer (Nanodrop). We considered samples that showed the absorbance ratio 260/280 at 1.8–2.0 and for 260/230 at 1.7–2.2. The Life&Brain company performed the genotyping analysis.

The Infinium Global Screening Array-24 v3.0 data were processed using the Illumina GenomeStudio 2.0 software to obtain information about chromosomes, positions, SNP type, total signal intensities (R) and B allele frequencies (BAF) for all probes in each sample. All probes referred to sex chromosomes (X, Y, XY), chromosomes 0 and MT, as well as probes with position 0, were not included in the analysis. All probes with unavailable values for R and BAF were removed. In addition, probes identified with SNP type equal to Indel or Deletion were ignored. The median (or mean) value for R and BAF in case of more than two (equal to two) duplicate probes was then computed. LogR values were determined by multiplying the base-2 logarithm of R by a parameter gamma set to 0.55.

LogR values were then adjusted for GC binding artifacts with ascat.create GCcontentFile.R [36]. ASMULTIPCF [37] algorithm was run with penalty parameter lambda set to subsequent values (from 70 to 150, in increments of 10) with LogR and BAF coming from clone(s) and fibroblast referred to the same patient as input data and the analysis was performed with the ASCAT (Allele-Specific Copy number Analysis of Tumors) R package [v3.1.2]. The results were the allele-specific copy number profile of each clone and fibroblast, as well as estimates of ploidy and cellularity.

#### Plasmid

pLVX-UbC-rtTA-Ngn2:2A:EGFP was a gift from Fred Gage (Addgene plasmid # 127288; http://n2t.net/addgene:127288; RRID:Addgene_127288) [20].

#### Lentiviruses Generation

On Day 0, the HeBS solution was prepared (NaCl 280 mM (Sigma-Merck; S7653), Hepes-Na 50 mM (Sigma-Merck; H3784), Na2HPO4 1.5 mM (Fluka; 71644), pH adjusted to 7.05 with HCl 1 M) along with 2.5 M CaCl2. Additionally, 500 ml of medium without P/S was prepared. In a second 15 ml falcon tube, 1250 µl of HeBS was vortexed at maximum speed, followed by the addition of the DNA-Ca2 + mix dropwise (one drop per second) while vortexing. The mixture was allowed to stand for 20 min at room temperature. The mixture was then added dropwise to the plates while gently moving them back and forth, followed by overnight incubation (12–16 h). On Day 3, the medium was changed (16 ml per plate), and the plates were returned to the incubator with complete medium for 36–48 h. The cell culture supernatant was then transferred to 50 ml falcon tubes, sealed with Parafilm, and centrifuged at 500 g for 4 h and 40 min at 4 °C using an Optima L-90 K Coulter centrifuge at 27,000 rpm. The next day, the pellets from three tubes were resuspended and pooled. Aliquots of 5 µl were made, and pre-cooled 0.5 ml Eppendorf tubes were used for storage at −80 °C [38].

#### Lentiviral Infection

At day −2, in a 6 well 50.000 single cells iPSC were plated then at day 0 cells were infected with 1 MOI of Lentivirus generated with pLVX-UbC-rtTA-Ngn2:2A:EGFP vector (Addgene plasmid, # 127288). The next day Lentivirus was removed and on day 2 we started the puromycin selection with increases doses, identified previously testing different puromycin concentration for each cell line, for 5 days.

#### FACS Sorting

Doxycycline (4 µg/mL) was added on day 6 for overnight induction of GFP expression the sorting experiment was performed the following day. Cells were treated with 0.05% Trypsin solution (Gibco, 25050014) after a single wash with HBSS. Cells were incubated at 37 °C for 3 min, followed by the addition of DMEM/F12 (Gibco, 11330057) and 10% FBS solution to neutralize the Trypsin activity. The cell suspension was then centrifuged at 500 g for 5 min. Subsequently, the cells were resuspended in HBSS with 5% FBS and passed through a 70 µm cell strainer (Greiner easy strainer 542040) to obtain a single-cell suspension. FACS was performed using the BD FACSymphony S6 cell sorter to collect GFP-positive cells. A small quantity of the collected cells was then plated into 96-well plates using the Automated Cell Deposition Unit (ACDU), seeding one cell per well to establish a clonal system. All FACS procedures were conducted under low-pressure conditions with a 100 µm nozzle. After sorting, pooled cells were centrifuged at 2000 rpm for 5 min and subsequently plated in Stemflex medium containing RI and Primocin (1:500) (InvivoGen; ant-pm-05). The next day, the rock Inhibitor was removed, and the iPSC-NGN2 cells were expanded.

#### iGluNeurons Differentiation

In order to generate induced Neurons (iGluNeurons) we employed a two-step protocol outlined in the timeline of Fig. 3A. Briefly, 300.000 single iPSC stable-NGN2 cells were plated in Geltrex coated 6 wells plate (counted using the automated cell counter Countess 3, Thermofisher), then cells were cultured with pre-differentiation media (composition) for 3 days. After 3 days, we cryopreserved the NPC using the iNeurons freezing solution [(80% KnockOut Serum Replacement (Gibco, 10828028) and 20% DMSO (Sigma, D2650)] and half solution of pre-differentiation media. After thawing, neuroprogenitor iGluNeurons were plated respectively on Cytoview 48 well (M768-tMEA-48B BLACK) for MEA recording, on petri dish (3 cm of diameters) for patch-clamp recordings, and on IBIDI (Twin Helix, 80806) for immunostaining assay. Previously, all supports were coated with Poly-L-ornithine hydrobromide (PLO) 50 µg/mL (Sigma-Aldrich, P3655) overnight, then washed three times with UltraPure water (Invitrogen, 10977049) and then coated with Laminin (10 µg/mL) (Sigma_Merck; L2020) in DMEM/F12 for 2 h at 37 °C. Then we plated dropwise 20 uL of iGluNeurons and primary rat astrocytes (ratio 1:3) with density of 1200 cell/mm2. Then iGLuNeurons were cultured for 56 days with two half media change per week, using Differentiation Media composed by Neurobasal medium (Gibco, 21103049) with B-27 2% (Gibco, 17504044), BDNF 10 ng/mL (Thermofisher; # 450–02), NT-3 10 ng/mL (Thermofisher; #450–03), Laminin 10 ng/mL and Doxycycline 4 ug/mL (that was removed after 14 days).

#### Patch-Clamp Recordings

Whole-cell patch clamp recordings in both voltage- and current clamp mode were performed using an EPC-10 amplifier (HEKA) and pulled borosilicate pipettes with a resistance of 3–5 MΩ. In each experiment, membrane capacitance, membrane and access resistance were routinely measured at the beginning of each recording (after about 2 min from establishing a GΩ seal) using the automatic compensation circuitry of the EPC-10 amplifier. Data acquisition was performed using PatchMaster program (HEKA). Only cells with access resistance of < 15 MΩ were considered for further analysis. All recordings have been performed at room temperature (22–24 °C). Both voltage- and current-clamp recordings were carried out using the following extracellular Tyroide solution containing (in mM): 140 NaCl, 2 CaCl 2, 1 MgCl 2, 4 KCl, 10 Glucose, 10 HEPES (pH 7.3 with NaOH). To avoid contamination ascribable to the synaptic transmission, D-AP5 (50 µM), CNQX (10 µM), CGP55845 (10 µM) and bicuculline (30 µM) were daily added to the external solution to block NMDA, non-NMDA, GABA A and GABA B receptors, respectively. The patch pipette was filled with the following intracellular solution (in mM): 126 K-Gluconate, 4 NaCl, 1 MgSO4, 0.02 CaCl 2, 0.1 BAPTA, 15 Glucose, 5 HEPES, 3 ATP, 0.1 GTP (pH 7.2 with KOH). Analysis of firing activity [39, 40]: spontaneous firing activity was recorded at the threshold potential of −40 mV while induced firing activity was assessed by the injection of a linear gradient of depolarizing current (Δ = 10 pA, duration of 500 ms). The rheobase was measured as the minimum amount of current needed to evoke at least one action potential. The amplitude of the action potential was calculated as the difference between the action potential peak and the threshold value. Instantaneous and mean firing frequency were determined at 100 pA of injected current. To be precise, the mean firing frequency was estimated as the ratio between the number of action potentials elicited after the injection of 100 pA of depolarizing current and the time interval between the first and last action potential. Instantaneous firing frequency was measured as the reciprocal value of the time interval occurring between the first two evoked action potentials after the injection of 100 pA of depolarizing current. Analysis of Na + currents: the Na + current density was assessed as the ratio between the peak inward current recorded at each depolarizing step and the cell capacitance (pA/pF).

#### Micro-electrode Array Recordings

The neuronal cultures were plated on commercial 48-well devices (CytoView MEA plate, Axion BioSystems, Atlanta, GA, USA). The devices integrate 16 electrodes for each well characterized by 50 μm in electrode size and arranged in a 4 × 4 grid (electrode spacing 350 μm). The electrophysiological recordings were performed with the Maestro Original (Axion BioSystems, Atlanta, GA, USA), once a week (± 2 days), from DIV 7 until DIV 56. Briefly, we let the cultures settle for 3 min at 37 °C. We performed the electrophysiological recordings of the spontaneous activity for 8 min sampled at 12.5 kHz. Data were collected applying a band pass filter, which high-pass-filter and low-pass-filter cutoff were set to 200 Hz and 3000 Hz, respectively. Off-line data analyses were performed by exploiting the Axion Software (Axion BioSystems, Atlanta, GA, USA) and custom-made in-house codes developed in MATLAB (The Mathworks, Natick, MA, USA). Briefly, the spike detection was performed with a hard-threshold detection which threshold was set to five times the standard deviation of the baseline noise. A channel was considered active whether its MFR was greater than 0.1 spikes/s [41]. From the spike detection, we extrapolated the MFR and the percentage of active channels. Moreover, based on the MFR trend from DIV 7 to DIV 56, we evaluated the maximum reached from the curve (i.e., DIV 49). We then performed a filtering of the dataset: we excluded from further analysis the wells with less than 10 over 16 active electrodes at DIV 49 to avoid considering cultures with no electrophysiological activity. Afterwards, the burst detection was performed based on the logarithmic ISI distribution [42, 43]. Bursts were detected by setting a threshold of the minimum number of spikes belonging to a burst equal to 5 spikes. Channels with a MBR lower than 0.4 bursts/min were excluded from the bursting analysis [44]. From the burst detection, we extrapolated the MBR, the BD, and the percentage of RS. Lastly, we defined an event as network burst if the activity was composed by consecutive bursts within a 100 ms window recruited from at least 30% of the channels. From the network burst detection, the NBR was computed. Neuronal network activity was recorded following acute chemical administration (APV 75 µM, CNQX 35 µM, and BIC 30 µM) at DIV 49. In the text, the values related to the electrophysiological features are reported as mean ± standard deviation.

#### Immunostaining

Cells were fixed for 10 min with 4%(v/v) paraformaldehyde (PFA) and 4% (w/v) sucrose, then cells were washed 3 times with PBS and permeabilized with 0.2% Triton X-100 in PBS for 10 min, followed by three washes with PBS. The following steps were performed using two different protocols: the nuclei markers protocol and the synaptic markers protocol. Nuclei Marker Protocol: a blocking solution (0.2% Triton X-100 and 10% FBS in PBS) was added for 45 min. Subsequently, primary antibodies were diluted in the incubation solution (0.2% Triton X-100 and 5% FBS in PBS) and added for overnight incubation. Four distinct markers for undifferentiated state were used: SOX2 (Millipore; AB5603; 1:500), NANOG (R&D Systems; AF1997; 1:500), OCT3/4 (R&D Systems; AF1759; 1:40), and SSEA-4 (Abcam; 16,287; 1:200). Secondary antibodies were used at a dilution of 1:500. Synaptic Marker Protocol: a blocking solution (10% FBS in PBS) was added for 45 min. Subsequently, primary antibodies were diluted in the incubation solution (5% FBS in PBS) was added for overnight incubation. The following antibodies were used: Ankyrin G (Santa Cruz; sc-28561; 1:200), MAP2 (Sigma; M9942; 1:200 and Synaptic Systems; 188003; 1:200), SMI312 (Biolegend; 837904; 1:200), Synapsin 1/2 (Synaptic Systems; 106004; 1:100), Synaptophysin (Millipore; MAB5258; 1:100), Tubulin beta III (Millipore; MAB1637; 1:200), NeuN (Millipore; MAB377; 1:200), PAX6 (Invitrogen; MA1-109; 1:200), vGLUT1 (Millipore; AB5905; 1:400) and Nestin (Invitrogen; MA1-5840; 1:500).

The next day, for both protocols, cells were washed three times with PBS for 5 min, followed by the addition of the secondary antibody Goat anti-Mouse IgG, IgM (H + L) Secondary Antibody, Alexa Fluor™ 488 (Invitrogen;Catalog # A-10680); Goat anti-Rabbit IgG (H + L) Cross-Adsorbed Secondary Antibody, Alexa Fluor™ 488 (Invitrogen; Catalog # A-11008); Goat anti-Mouse IgG (H + L) Cross-Adsorbed Secondary Antibody, Alexa Fluor™ 568 (Invitrogen; Catalog # A-11004; Goat anti-Rabbit IgG (H + L) Cross-Adsorbed Secondary Antibody, Alexa Fluor™ 594 (Invitrogen; Catalog # A-11012), Donkey anti-Goat IgG (H + L) Cross-Adsorbed Secondary Antibody, Alexa Fluor™ 488 (Invitrogen; Catalog # A-11055 (1:500) in the same dilution buffer for 1 h in the dark. The secondary antibody was then removed, and Hoechst (1:300) was added for 15 min, followed by three washes with HBSS for 5 min.

#### Statistical Analysis

All experiments were replicated at least three times. Data are expressed as means ± standard error of the mean (SEM) for the number of independently differentiated iPSCs lines unless differentially stated. The normal distribution of data was assessed using the D'Agostino-Pearson's normality test. The unpaired or paired two-tailed Student’s t-test was used to compare two normally distributed sample groups. To compare two-sample groups that were not normally distributed, the Mann–Whitney’s U-test was used. For micro-electrode array recordings, the statistical analyses were performed using the Wilcoxon test (non-parametric, for paired data), and p-values were adjusted using the Benjamini-Hochberg correction. Statistical analysis was carried out using Prism (GraphPad Software, Inc.)

## Supplementary Information

Below is the link to the electronic supplementary material.

Supplementary file1 (PDF 5164 KB)

## Data Availability

All authors ensure that all data and materials support the published claims and comply with field standards.
