# Supplementary material for: Optimization of Transcription Factor-Driven Neuronal Differentiation from Human Induced Pluripotent Stem Cells for Disease Modelling and Drug Screening
Source: Stem Cell Rev Rep. 2025 Jan 31;21(3):816–33. doi: 10.1007/s12015-025-10845-4 (PMC11965252; doi:10.1007/s12015-025-10845-4)

SUPPLEMENTARY MATERIALS

Supplementary Figure 1. Pool/single clone sorting strategies and pluripotency quality controls after FACS sorting

(A) Representative dot plots depicting the sorting strategy. The upper panels show the process starting with doublet exclusion, followed by the selection of the GFP-positive cell population (left dot plot), which corresponds to the median fluorescence value (graph on the right). This approach sorts the largest number of cells with the same characteristics, as supported by a density plot (middle panel). (B) Representative dot plots showing the details of single-cell deposition. This phase is also useful for verifying the purity level of the previous sorting, as illustrated by the exact location of each of the 96 plated cells (bottom panels). (C) Representative immunostaining of undifferentiated state markers: NANOG, SOX2, OCT3/4, and SSEA4 after FACS sorting (D) Quantification of each undifferentiated state markers shown as percentages of DAPI-positive nuclei for all IPSCs lines (means ± SEM). (E) Representative flow cytometry dot plot for GFP expression in iPSC-NGN2 (5 split after sorting) induced with or without doxycycline induction. (F) Graph of flow cytometry data expressed as percentages of NGN2/GFP positive cells of all 7 iPSCs lines tested along the study (means ±SEM).

Supplementary Figure 2. Assessment of glutamatergic identity and patch clamp analysis of DIV56. (A) Representative immunostaining of vesicular glutamate transporter 1 (vGlut1) at DIV 35-42 as a marker for the glutamatergic neurons (scale bar 10 µM). (B-E) Scatter plots of: (B) normalized Mean Firing Rate (MFR); (C) normalized amount of Bursting Electrodes (BE); (D) normalized Random Spikes (RS); and (E) normalized Network Bursting Rate (NBR) during the spontaneous and chemically modulated activity. The normalization of the data was performed with respect to the values of the same feature during the spontaneous activity. In the scatter plots, data are represented with the mean (horizontal line) and the standard error of the mean (error bar). (F) In vitro whole cell current clamp recording of the action potential spontaneous activity recorded at the subthreshold potential of -40 mV. (G) *In vitro* whole cell current clamp recording of the action potential firing activity elicited with the injection of depolarizing currents. (H) Representative whole cell current families recorded from iGluNeurons at DIV56 of in vitro differentiation. Cells were clamped at -70 mV.

Supplementary Table 1

DIV	7			14			21			28			35			42			49			56		
7	AE	MFR		-	-	-	0.0004	5.9 e-05	5.1 e-05	0.0001	5.1 e-05	5.1 e-05	4.8 e-05	4.7 e-05	5.9 e-05	4.8 e-05	4.8 e-05	5.1 e-05	4.8 e-05	4.7 e-05	5.1 e-05	0.0001	4.7 e-05	5.1 e-05
14	0.009	0.0004					0.0004	5.9 e-05	5.1 e-05	0.0001	5.1 e-05	5.1 e-05	4.8 e-05	4.7 e-05	5.9 e-05	4.8 e-05	4.8 e-05	5.1 e-05	4.8 e-05	4.7 e-05	5.1 e-05	0.0001	4.7 e-05	5.1 e-05
21	7.1 e-05	0.0004		0.0005	0.03					0.048	0.02	0.9	0.0008	0.006	1	0.003	0.03	0.9	0.0002	0.002	0.9	0.002	0.001	0.7
28	7.1 e-05	0.0004		9.8 e-05	0.002		0.2	0.08					0.03	0.6	0.9	0.04	0.7	0.9	0.002	0.08	0.9	0.012	0.003	0.9
35	7.1 e-05	0.0004		9.4 e-05	0.0006		0.004	0.0006		0.051	0.0017					0.3	1	0.9	0.06	0.3	0.9	0.3	0.08	0.8
42	7.1 e-05	0.0004		9.4 e-05	0.0004		9.8 e-05	0.0004		0.0003	0.0006		0.07	0.1					0.7	0.3	0.9	0.7	0.1	0.9
49	7.1 e-05	0.0006		9.8 e-05	0.0017		0.00012	0.003		0.00015	0.013		0.011	0.09		0.2	0.6					0.4	0.1	0.7
56	7.4 e-05	0.03		0.0001	0.6		0.00015	0.4		0.0003	0.3		0.011	0.2		0.1	0.02		0.6	0.02		MBR	RS	BD

P-values related to the comparison of the firing- and bursting- related features AE, MFR, MBR, BD, and RS) at each DIV adjusted with the Benjiamini-Hochberg correction.

Supplementary Table 2

DIV	7	14	21	28	35	42	49	56
7								
14	-							
21	-	-						
28	0.02	0.02	0.1					
35	0.0004	0.0004	0.002	0.03				
42	0.002	0.002	0.01	0.1	0.5			
49	0.0002	0.0002	0.0002	0.0002	0.0006	0.2		
56	0.0002	0.0002	0.001	0.003	0.003	0.2	0.9	

P-values related to the comparison of the network bursting related feature, i.e., NBR, at each DIV adjusted with the Benjiamini-Hochberg correction.

Supplementary Table 3

DIV	7	14	21	28	35	42	49	56
AE (%)	0 ± 0	10 ± 10	36 ± 13	46 ± 13	65 ± 22	82 ± 15	92 ± 9	93 ± 11
MFR (spikes/s)	0.00 ± 0.00	0.30 ± 0.13	0.60 ± 0.30	0.95 ± 0.41	1.90 ± 0.44	2.72 ± 1.00	3.24 ± 1.94	1.12 ± 1.53
MBR (bursts/min)	0.00 ± 0.00	0.00 ± 0.00	1.41 ± 1.23	3.19 ± 2.10	5.40 ± 2.02	7.43 ± 4.60	8.55 ± 3.74	6.70 ± 3.11
RS (%)	100 ± 0	100 ± 0	66 ± 18	42 ± 12	35 ± 21	37 ± 25	27 ± 17	17 ± 13
BD (ms)	0 ± 0	0 ± 0	285 ± 84	310 ± 105	284 ± 47	335 ± 124	293 ± 95	425 ± 219
NBR (NBs/min)	0.00 ± 0.00	0.00 ± 0.00	0.00 ± 0.00	0.20 ± 0.32	0.97 ± 0.95	3.93 ± 4.31	5.53 ± 2.04	5.49 ± 2.53

Average values from MEA recordings (mean values ± standard deviation) for each DIV of Active Electrodes (AE), Mean Firing Rate (MFR), Mean Bursting Rate (MBR), Random Spikes (RS), Burst Duration (BD), and Network Bursting Rate (NBR) at each DIV.

Suppl FIGURE 1

A **Pool sorting**

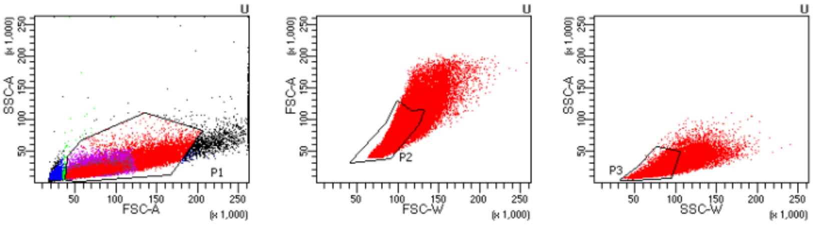

Population	#Events	%Parent	GFP B_530... Mean	GFP B_53... Median
P1 AND P2 AND P3	####	78.6	8,894.99	96.21
GFPsort	11,592	14.8	19,967.63	19,647.68
Neg	####	####	85.21	66.83
GFP_Pos	27,667	35.2	25,108.71	20,790.29
Q3	####	####	####	####
Q4	####	####	####	####
Singlecell_Sort	8,563	10.9	20,780.57	20,540.73

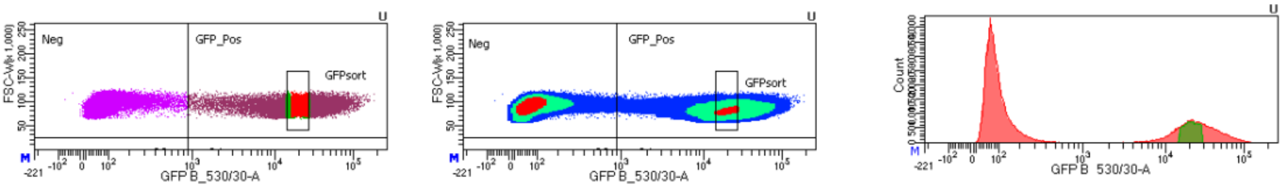

B **Single clone sorting**

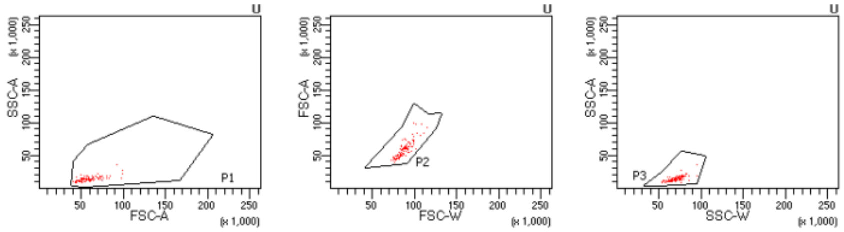

Population	#Events	%Parent	GFP B_530... Mean	GFP B_53... Median
P1 AND P2 AND P3	####	100.0	21,052.27	20,816.54
GFPsort	96	100.0	21,052.27	20,816.54
Neg	####	####	####	####
GFP_Pos	96	100.0	21,052.27	20,816.54
Q3	####	####	####	####
Q4	####	####	####	####
Singlecell_Sort	93	96.9	20,879.15	20,726.39

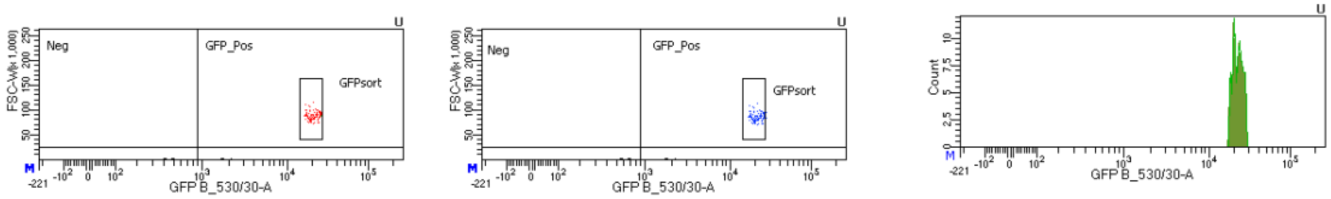

C

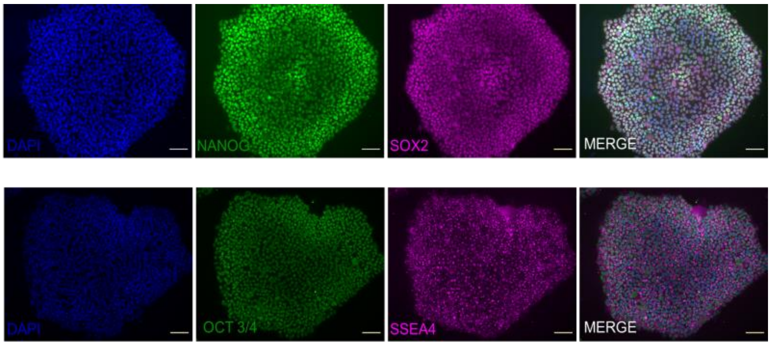

D

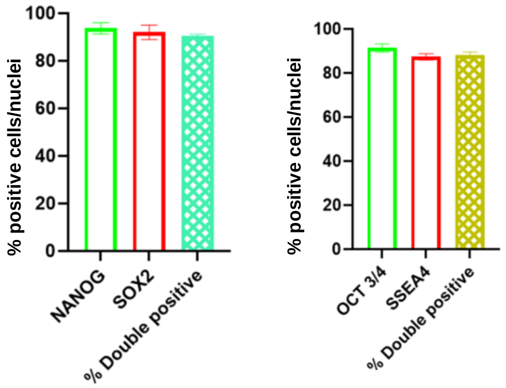

E

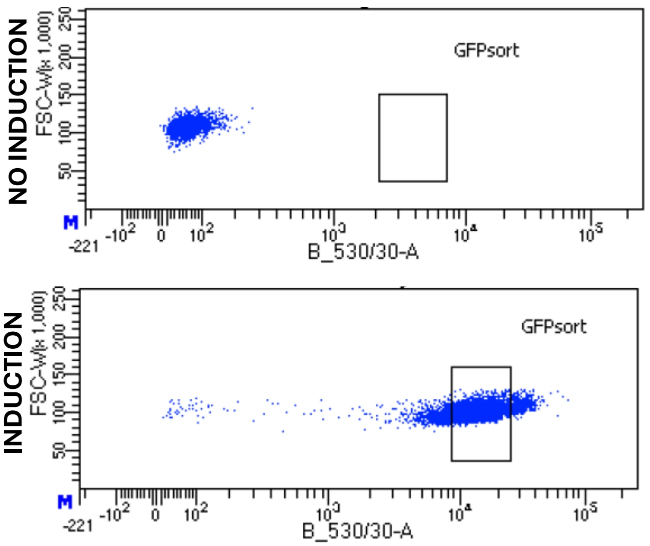

F

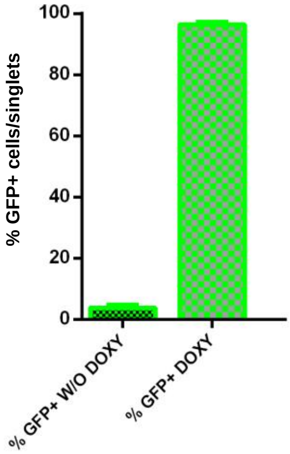

Suppl FIGURE 2

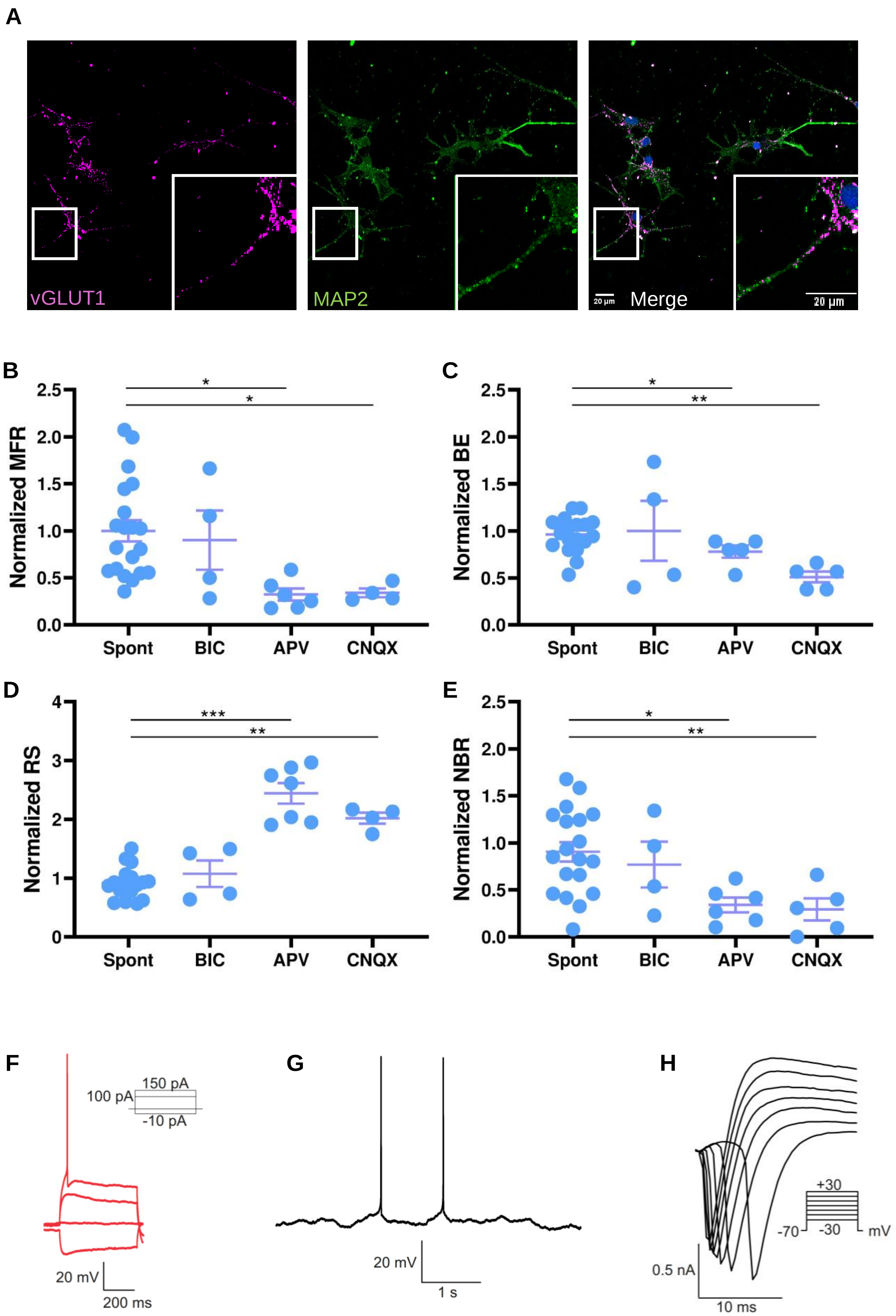

Supplement: Supplementary file 1 — Supplementary file1 (PDF 5164 KB) [file 12015_2025_10845_MOESM1_ESM.pdf]
